# Supplementary material for: Hemodynamic and Respiratory Changes following Prone Position in Acute Respiratory Distress Syndrome Patients: A Clinical Study
Source: J Clin Med. 2023 Jan 18;12(3):760. doi: 10.3390/jcm12030760 (PMC9917844; doi:10.3390/jcm12030760)
Supplement: Supplementary file 1 [file jcm-12-00760-s001.zip › jcm-2109293-supplementary.pdf]

Supplemental Information

Supplementary Material S1

Materials and methods

Settings in semirecumbant baseline (SRBAS) and prone posture

Our institution's protocol for mechanical ventilation was based in the principles of the ARDSnet protocol. Briefly, respiratory rate was set to achieve $PaCO_2$ 40-90 mmHg and pH >7.15, at a maximum set respiratory rate 35bpm. Consequently, in case of change in the need for oxygenation and/or ventilation during the prone position, FiO_2 was allowed to be titrated first, and secondarily PEEP, in order to achieve PaO_2:55-80 mmHg or SpO_2:88-95%. During measurements and when needed for better patient-ventilator coordination, patients were additionally paralyzed with bolus injections of cisatracurium (10 to 20mg). Patients were ventilated in the prone posture for at least 18 hours and were then turned to the supine position. Scheduled measurements were at SRBAS (semi recumbent baseline), just before prone position, at 1h and 18h of prone positioning. We aimed to keep ventilator settings unchanged for at least 3 hours before and during position changing if possible.

Patients were positioned flat, on air mattresses, with a dynamic alternating cell design but nonetheless additional cushions were used under the face to protect the eyes in the prone posture. Cushions were also used under the chest and the pelvis to permit free abdominal movement when prone. In the prone position arms were laid parallel to the body and attention was paid to avoid any non-physiologic movement of the limbs during posture changes. Tracheal suctioning was performed by means of an in line suction catheter (Ballard Trach Care, 16 Fr, Ballard Medical Products, Draper UT) permitting suctioning without discontinuation of the ventilator circuit. No bronchial suctioning was allowed at least half an hour before each set of measurements.

Measurements

Pulmonary artery catheter (PAC) data included measurement of cardiac output (CO), right ventricular (RV) ejection fraction (EF), RV end-diastolic volume (EDV), pulmonary artery pressure (PAP), pulmonary artery occlusion pressure (PAOP), oxygen saturation of mixed venous blood (SvO2). A Swan-Ganz Continuous CO/EDV/RVEF Thermodilution Catheter (7.5-Fr, Edward's Lifesciences Germany GmbH) and disposable pressure transducers were used. Transducers were calibrated for zero pressure at atmospheric pressure in the mid-axillary line, forth intercostal space. With each position change pressure transducers were repositioned and re-calibrated. The PAOP was obtained after inflating the balloon with 1.5 ml air. Criteria for adequate wedge position of the pulmonary artery catheter tip were a phasic waveform synchronized to the electrocardiogram, an end expiratory PAOP (eePAOP) value less than the end- expiratory diastolic PAP value (eedPAP), and a similar increase during inspiration of both PAOP and diastolic PAP (dPAP) validating that the occluded pulmonary artery catheter tip was not in segments of the lung reflecting zone 1 or zone 2 conditions, according to a method described previously [1]. All measurements of pressures were obtained in the semi recumbent position (SR_{BAS}) with a head tilt of $>30^0$ when supine but in horizontal position when prone. Right ventricle end-diastolic volume (EDV) was computed by the Vigilance monitor that was simultaneously connected to the pulmonary artery catheter and the ECG monitoring system permitting computation of ejection fraction based on thermodilution methods. Subsequently, continuous stroke volume (SV), end systolic (ESV) and end diastolic (EDV) volume measurements of the right ventricle were derived from the ejection fraction (EF) and cardiac output (CO) measurements as follows: SV=CO/HR, EDV= SV/ EF, ESV= EDV- SV. Calculation of shunt fraction was done using the shunt equation: $Qs/Qt=(CcO_2-CaO_2)/(CcO_2-CvO_2)$ assuming for the calculation of CcO_2 (=Oxygen content of pulmonary capillary blood) that PcO_2 (=partial pressure of capillary blood O_2) equilibrates with PAO_2 (=partial pressure of alveolar air O_2); therefore the alveolar air equation was used to calculate CcO_2, and that the ScO_2 (saturation of pulmonary capillary hemoglobin) is 100% (as this is determined before the anatomical shunts mix with the oxygenated blood). The equation to calculate PAO_2 was PAO_2= $(Paw-P_{H2O}) \times FiO_2 - (PaCO_2/RQ)$ (RQ=0.8). Plateau pressure and PEEP were measured after end-inspiratory (2 sec) and end-expiratory (2 sec) occlusions, respectively. Tidal volume was measured by the means of the expiratory flow transducer of the ventilator. The static compliance of the respiratory system was calculated as follows: Cst,rs= tidal volume/(plateau pressure- PEEP).

References

1 . Teboul JL, Besbes M, Andrivet P. A bedside index accessing the reliability of pulmonary occlusion pressure during mechanical ventilation with positive end-expiratory pressure. J Crit Care 1992; 7:22-29.

Table S1. Significant relationship between the Oxygenation Index at 18 hours at prone posture (OI_{18p}) and clinical, hemodynamic and respiratory indices.

	correlation coefficient*	P value
**Baseline values**		
**OI**	0.52	0.028
**$P(v\text{-}a)CO_2$**	0.54	0.038
**$P(v\text{-}a)CO_2/C(a\text{-}v)O_2$**	0.51	0.031
**ARDS score**	-0.69	0.021
**PO_2/FiO_2**	-0.50	0.035
**1hProne values**		
**PO_2/FiO_2**	-0.50	0.035
**OI**	0.52	0.028
**$P(v\text{-}a)CO_2$**	0.51	0.03
**18hProne values**		
**PCO_2**	0.57	0.013
**$\dot{Q}s/\dot{Q}t$**	0.72	0.001
**dPAP**	0.47	0.049
**TPG**	0.50	0.038
**EDVi**	0.51	0.028
**Changes (Δ) from SRBAS to 1hProne**		
**ΔSVR**	-0.51	0.030
**ΔVO_2**	0.49	0.041
**ΔCI%**	0.48	0.044
**$\Delta PO_2/FiO_2$**	-0.64	0.003

* Pearsons r, otherwise is indicated. Definition of abbreviations: ΔCI%=percent change of cardiac index; dPAP=diastolic pulmonary artery pressure; EDVi= Right ventricle end diastolic volume index; PaCO2=Arterial partial pressure of CO2; PaO2/FiO2 =ratio of arterial partial pressure of O2, to inspired oxygen fraction; P(v-a)CO2= veno-arterial carbon dioxide gradient; P(v-a)CO2 /C(a-v)O2= ratio of venoarterial carbon dioxide tension gradient over the arteriovenous oxygen content gradient; $\dot{Q}s/Qt$ = pulmonary shunt to cardiac output fraction; SRBAS=baseline semirecumbent posture; SVR= systemic vascular resistance; TPG=MPAP-WP=difference between mean pulmonary artery pressure and occlusion (wedge) pressure; VO2= oxygen consumption
